# Editorial: Stroke Care Networks in Disadvantaged Groups

**DOI:** 10.3389/fneur.2022.941095

**Published:** 2022-05-31

**Authors:** Candice Delcourt, Antonio Arauz, Miguel A. Barboza

**Affiliations:** ^1^The George Institute for Global Health, Faculty of Medicine, University of New South Wales (UNSW), Sydney, NSW, Australia; ^2^Department of Clinical Medicine, Faculty of Medicine, Health and Human Sciences, Macquarie University, Sydney, NSW, Australia; ^3^Stroke Clinic, Manuel Velasco Suárez Instituto Nacional de Neurología y Neurocirugía, Mexico City, Mexico; ^4^Hospital Dr. Rafael A. Calderon, Neuroscience Department, University of Costa Rica, San José, Costa Rica

**Keywords:** stroke, disadvantaged groups, health system, barrier, healthcare

Stroke is a leading cause of disability worldwide. Advances in acute care in the last 5 years have improved survival. However, this improvement has not been seen in every health care setting. There is a gap in care for disadvantaged patients whether the inequality is caused by country (low- and middle-income country), remoteness (rural vs. metropolitan), ethnicity (African American, Aboriginal, and Torres Strait Islanders), or socio-economic isolation (uninsured, low ages).

In this Research Topic, we aimed to understand disparities and barriers in accessing stroke care and to explore strategies that can improve care.

Gaps in stroke may arise from barriers to patients accessing existing care such as lack of insurance, place of residence, and education. Failure of systems to care may arise from lack of hospital expertise, poor infrastructure, staffing and resource limitations, absence of stroke care system pathways, protocols or guidelines and limited support from funding bodies, institutions, and government.

In the Philippines (Collantes et al.), there is limited government financing, large out-of-pocket costs, limited resources, limited workforce, no stroke awareness program, no program to target stroke risk factors and an unknown burden because of the absence of a national registry.

At the Instituto Nacional de Neurología y Neurocirugía Manuel Velasco Suárez (INNNMVS) stroke clinic in Mexico City (Cano-Nigenda et al.), there were delays in accessing the stroke facility because of presentation to other facilities, arrival by private car and low rates (<4%) of pre-hospital notification. Patient and carer interviews revealed limited awareness of stroke symptoms, degree of urgency, and the possibility of effective treatment.

Strategies proposed by the authors to improve stroke care included transition to universal healthcare and development of stroke care pathways based on World Health Organization recommendations. They also emphasized the importance of collecting stroke incidence data. The role of stroke incidence data in understanding gaps in stroke care was also highlighted by Balabinski et al. who published the protocol of a systematic review on stroke in Indigenous populations. Cano-Nigenda et al. highlighted the importance of public education, improvement of patient transfer networks and use of telemedicine. Challenges in the transition to digital health technologies often limit the use of telemedicine despite this being recognized as a means to improve stroke care in non-urban areas. Busti et al. reviewed the barriers and strategies for transition to digital health technologies. They highlighted measures to improve the transition including; integration of digital health technologies in medical education, recognition by medical societies, and funding for digital health technologies and practitioners using them.

The confirmation of the efficacy of endovascular therapy (EVT) in 2015 created additional challenges for non-urban centers and low-income countries. Only specialized centers with highly qualified practitioners can provide EVT. In Mexico (Gongora-Rivera et al.), a survey of Mexican endovascular neurologists showed that the main barriers in accessing EVT in private and public hospitals were the lack of health coverage for EVT by the National Health system, the cost of medical supplies for EVT, the inadequate recognition of stroke symptoms in the population and the low frequency of EVT requests in hospitals. In 90% of Mexican hospitals there was an out-of pocket cost and, in over half of them, this was over US$10,000. They emphasized the need for efficient use of public funding and stroke education campaigns.

Disparities in aspects of stroke care were highlighted by Chen et al. who reviewed the differences in the blood pressure management of acute stroke in the ENCHANTED trial. They showed that in China, patients with acute ischemic stroke were less likely to receive intravenous blood pressure lowering agents than in other parts of Asia, Western countries, and South America. The intravenous drugs used for lowering blood pressure varied between countries. Overall, blood pressure reduction and variability were found to be less intensive in China compared to other countries.

Identifying barriers is the first step in improving services. Additional steps include the development of regional stroke care plans as described by Martins et al. In the 2 years following the declaration of Granada, which delineated priorities to improve stroke care in Latin America, they showed an increase in public stroke awareness initiatives from 25 to 75%, new programs to encourage physical activity, and the implementation of strategies to identify and treat hypertension, diabetes, and lifestyle risk factors. The number of stroke centers providing intravenous thrombolysis increased. There was an increase in countries with stroke units. All countries had centers providing mechanical thrombectomy, but mostly restricted to a few private hospitals.

This regional stroke plan for Latin America is a model for other under-resourced environments.

The translation of recommendations or guidelines to clinical practice can be challenging. Novarro-Escudero et al. described successful implementation of a private primary stroke center in Panama. Their methodology included: provision of guidelines and best practices review; site surveys to determine the status of current stroke care, increased resources, staff and logistics; nomination of a multidisciplinary task force to create policy, procedures and workflows; an intensive education program, and a quality improvement process to evaluate performance before accrediting a stroke center.

Understanding the regional and local barriers, and acknowledging the gaps and needs in different socioeconomic settings, is a challenge that includes not only individual healthcare givers, but is also a responsibility that governments and health care delivery systems need to embrace to improve stroke care delivery to those most in need.

## Author Contributions

CD wrote the first draft. All authors provided a critical review and approved the manuscript prior to submission.

## Conflict of Interest

The authors declare that the research was conducted in the absence of any commercial or financial relationships that could be construed as a potential conflict of interest.

## Publisher's Note

All claims expressed in this article are solely those of the authors and do not necessarily represent those of their affiliated organizations, or those of the publisher, the editors and the reviewers. Any product that may be evaluated in this article, or claim that may be made by its manufacturer, is not guaranteed or endorsed by the publisher.

